# Exploring Nepicastat Activity: Beyond DβH

**DOI:** 10.3390/ijms26094356

**Published:** 2025-05-03

**Authors:** Rafal Jas, Marta Bauer, Błażej Grodner, Weronika Kończak, Karolina Frączek, Anna K. Laskowska, Małgorzata Milczarek, Wojciech Kamysz, Patrycja Kleczkowska

**Affiliations:** 1 Maria Sklodowska-Curie Medical Academy in Warsaw, 03-411 Warsaw, Poland; rafal.jas@onet.eu; 2 Department of Analytical Chemistry, Faculty of Pharmacy, Medical University of Gdansk, 80-416 Gdansk, Poland; marta.bauer@gumed.edu.pl; 3 Chair and Department of Biochemistry and Pharmacogenomics, Faculty of Pharmacy, Medical University of Warsaw, 02-097 Warsaw, Poland; blazej.grodner@wum.edu.pl (B.G.); s088887@student.wum.edu.pl (W.K.); 4 Department of Pharmacotherapy and Pharmaceutical Care, Faculty of Pharmacy, Medical University of Warsaw, 02-097 Warsaw, Poland; karolinapawlik89@gmail.com; 5 Department of Pharmaceutical Microbiology and Bioanalysis, Medical University of Warsaw, 02-106 Warsaw, Poland; anna.laskowska@wum.edu.pl; 6 Department of Biomedical Research, National Medicines Institute, 00-725 Warsaw, Poland; m.milczarek@nil.gov.pl; 7 Department of Inorganic Chemistry, Faculty of Pharmacy, Medical University of Gdansk, 80-416 Gdansk, Poland; wojciech.kamysz@gumed.edu.pl

**Keywords:** nepicastat, efficacy, biological activity, antinociception, acetylcholinesterase inhibition

## Abstract

Recently, an old drug, disulfiram, has been shown to reduce cocaine intake by inhibiting dopamine beta (β)-hydroxylase. Its effectiveness was also reported in opioid treatment, as disulfiram attenuated morphine-induced tolerance and dependence. A similar mechanism of action was evident in a selective inhibitor of DβH, nepicastat, particularly in the aspect of cocaine-seeking behavior. Hence, the objective of this study was to verify whether or not nepicastat reproduces disulfiram activity in pain reduction. Moreover, determination of its likely biological effects resulting from interactions with targets other than DβH has been given, in particular acetylcholinesterase. As was found, nepicastat was characterized by the absence of desired antinociceptive activity, though its co-administration with morphine resulted in a dose- and time-dependent enhancement of morphine-induced analgesic effect and attenuation of tolerance. Similarly, nepicastat was found to manifest antimicrobial potency against selected bacterial strains, although the effect was found to be weak. Intriguingly, this compound interacted with acetylcholinesterase through inhibition of its activity. These results clearly indicate nepicastat as a potent molecule that exhibits various biological effects. This, in turn, suggests its possible application in pathological conditions that still require effective treatment.

## 1. Introduction

Dopamine beta (β)-hydroxylase (DβH), located in the brain, adrenal medulla, heart, and other sympathetically innervated organs, serves as an enzyme responsible for the biosynthesis of noradrenaline (NA) from dopamine (DA). Hence, its insufficient level can impair the production of the above-mentioned substances and thus contribute to the occurrence of the disease. In fact, this process is critical for the proper functioning of the sympathetic nervous system and the regulation of various physiological functions. DβH is primarily expressed in the brain, adrenal medulla, and other sympathetically innervated tissues [1,2,3,4,5]. By catalyzing the hydroxylation of dopamine, DβH facilitates the biosynthesis of noradrenaline, which is essential for regulating blood pressure, stress responses, and cognitive processes such as attention, arousal, and memory. Individuals with DβH deficiency are vulnerable to developing a range of serious health complications, including hypotension, hypoglycemia, and, in some cases, even Parkinson’s disease [1,3]. In contrast, elevated DβH expression has also been linked to cardiovascular diseases, such as hypertension, as well as a range of neuropsychiatric disorders, including depression, likely due to excessive production of NA [4,5,6,7].

Interestingly, a correlation was also found between pain and the amount and activity of the enzyme. Eshel et al. showed that a high activity of DβH was associated with the occurrence of pain [8]. Similar results were also found in migraine patients [9,10]. In addition, an excessive increase of DβH-immunoreactive neurons was also detected in the mice spinal cord as a result of injury to nerves, thus strictly associating it with neuropathic pain [11].

In view of the above, targeting DβH could be a therapeutic option. In this context, DSF was one of the well-known drugs whose efficacy was attributed to the inhibition of DβH. However, another drug, nepicastat (NEP), also known as SYN-17 or RS-25560-197, which is characterized by significantly higher effectiveness as a DβH inhibitor compared to that of DSF and improved selectivity for DβH over other enzymes [12], has been proposed as a potential therapeutic agent. Importantly, NEP was found to reproduce some of the activities of DSF. Similar to DSF, it has therefore been suggested as a potentially useful remedy for the treatment of substance abuse, particularly for attenuating various aspects of cocaine- or alcohol-seeking behaviors, respectively [13,14,15,16]. NEP has also been reported to have beneficial cardiovascular effects in hypertension and congestive heart failure [17,18]. More recently, DSF has been reported to suppress pain and morphine withdrawal symptoms and to delay morphine-induced tolerance in laboratory animals [19]. The data and similarities between these two drugs prompted us to investigate whether or not NEP could also be useful in the treatment of opioid-induced side effects but also to verify if its spectrum of activity is not limited only to mitigating the effects of addictive substances, such as cocaine.

The aim of the study was to promote NEP as a potent agent with a plethora of beneficial effects, including analgesic activity. The current study also included estimation of possible interaction between NEP and acetylcholinesterase (AChE; EC 3.1.7) based on literature reports indicating that (i) AChE and dopamine (DA) were co-released from the neurons that degenerated in various neurodegenerative diseases, particularly Parkinson’s and Alzheimer’s diseases, and (ii) DβH activity strongly correlated with AChE [20,21]. Consequently, valuable insight into the characteristics of NEP was gained.

## 2. Results and Discussion

### 2.1. NEP Exhibits a Weak Antimicrobial Activity Depending on the Strain Used

Although the exact mechanism behind NEP’s antibacterial action is still unknown, the fact that it has been shown to inhibit enzymatic pathways in mammals suggests that it may also have an impact on similar targets in bacteria, such as membrane-associated proteins or oxidoreductases. It is necessary to conduct more mechanistic research.

In this aspect, preliminary studies suggested that NEP exhibited a modest antimicrobial activity, as some of the bacterial strains exposed to it were found to be sensitive (Table 1). Among the tested pathogenic bacteria, *S. aureus* was discovered to have the highest sensitivity to the NEP molecule already at a concentration as low as 64 µg/mL. However, this activity was much lower than that of the reference drug, ciprofloxacin. Other bacteria, in particular multidrug-resistant *P. aeruginosa*, did not prove to be a target for NEP.

Since there is no information on the possible mechanism by which NEP might have antibacterial efficacy, several factors are likely to influence its behavior. In fact, as presented by others, bacteria might appear as susceptible when the inoculum is standardized (10^5^ CFU/mL) but resistant when the inoculum size is increased [22,23,24]. In addition, it was suggested that the failure to produce the desired antimicrobial effect originates from the interaction of the drug with the medium used to grow the bacteria [25]. For this reason, we cannot completely conclude that the molecule does not show any activity. The study would need to be repeated by first verifying whether or not there is an inoculum phenomenon in the bacterial strains tested by using different concentrations of inoculum; second, increasing the range of strains tested to other types of bacteria or even fungi; and third and finally, determining the activity of NEP under in vivo conditions, thus allowing for gaining more realistic results.

### 2.2. NEP Is a Non-Hemolysis-Inducing Agent

**Figure 1:**
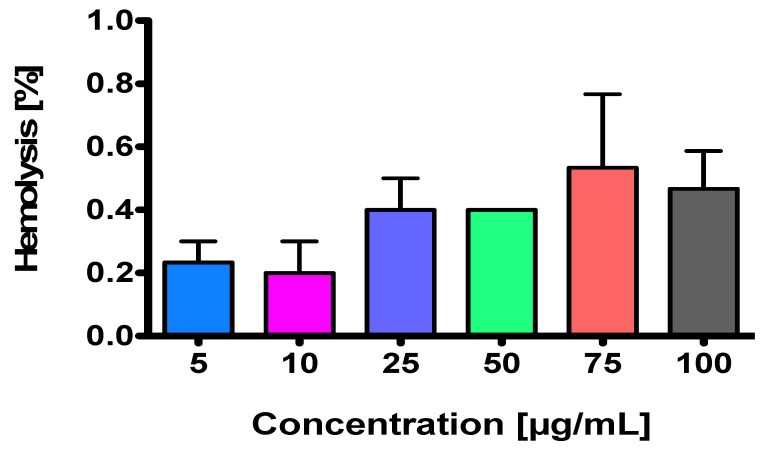
Hemolytic activity of NEP. One-way ANOVA followed by Bonferroni’s post hoc test revealed no significant differences between each dose used.

In vitro anti-hemolytic activity of NEP on sheep red blood cells was determined. The results indicated that the tested DβH inhibitor exhibited clinically acceptable hemolytic activity at each concentration used (Figure 1). In fact, desired hemolysis frequencies for the compound being a drug candidate should be <2% [26], and in the case of NEP, these values vary between 0.2% and 0.533%. Although it seems that the lysis of erythrocytes grows with an increase in NEP concentration, no statistical differences have been noted.

Obviously, these results should be confirmed using human erythrocytes. In fact, the species origin of blood used for hemolysis assays can affect the hemolytic response [27]. This can also include detergents and even distilled water that are commonly used to perform the assay.

### 2.3. NEP Exerts Activity Against Acetylcholinesterase

Since DβH activity was found to be highly correlated with AChE [21], we also examined whether or not a selective DβH inhibitor, NEP, can also affect AChE activity. Given that virtual screening methods have now been widely used in pharmaceutical research to identify the most promising drug candidates before conducting costly and lengthy experiments, we first used the freely accessible web tool MolPredictX. Another step was to determine NEP activity in vitro, which also helped us verify both the relevance and reliability of the in silico method.

**Figure 2:**
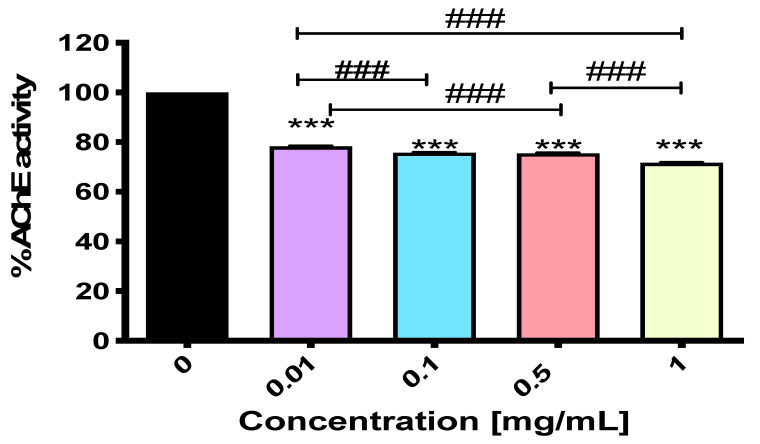
AChE-inhibitory activity of NEP. One-way ANOVA followed by Tukey’s post-hoc test revealed significant differences between each NEP concentration vs. control group (100% activity of AChE)—*** *p* < 0.001 and between each concentration (^###^ *p* < 0.001). No significant differences were noted between NEP administered at a concentration of 0.1 vs. 0.5 mg/mL.

As can be seen, according to the results obtained in the “virtual environment”, NEP showed some interaction with AChE (Table 2). Unfortunately, these results do not clearly indicate the nature of the tested compound, i.e., whether or not in this particular case it acts as an inhibitor or a stimulator of the enzyme. Importantly, our next experiment confirmed some modest activity of NEP against AChE, and this was found to be a stronger inhibition than stimulation of the enzyme. Indeed, NEP was able to dose-dependently inhibit AChE activity; however, no significant differences were found between its concentrations of 0.1 and 0.5 mg/mL (Figure 2). While NEP at 0.01 mg/mL led to AChE inhibition from 100% to 78.289 ± 0.090% with an inhibitory potency of 21.711%, an increased concentration (up to 1 mg/mL) improved its inhibitory activity fairly well. In fact, the percentage of enzyme inhibition was equal to 28.343%, as AChE activity dropped from 100% to 71.657 ± 0.072% (Figure 2). These results were statistically significant, with an F-value of *F*_4,10_ = 15,348.94 (*p* < 0.0001), indicating a highly significant difference between the groups.

As AChE has been shown to be one of the most important targets in Alzheimer’s disease (AD) [28], drugs with inhibitory effects (such as donepezil and rivastigmine [29]) have recently dominated the treatment of patients. This is also due to the fact that AChE inhibition was thought to affect the processing of amyloid precursor protein by preventing the formation of its toxic oligomeric form [30,31].

In our studies, we announced NEP for the first time as a potential compound that could positively affect AChE activity. While NEP exhibited weaker inhibitory activity compared to more established inhibitors like selective donepezil (which has an IC₅₀ value of 0.05 ± 0.06 μM for AChE inhibition) [32], this could be due to NEP’s weaker or more peripheral binding to the enzyme. In contrast, donepezil shows a stronger binding affinity to AChE in silico, as demonstrated by various molecular docking studies. For instance, one study reported a binding affinity of −9.33 kcal/mol when donepezil was docked into the AChE enzyme [33], while another study indicated a binding affinity of −9.8 kcal/mol in the same enzyme structure [34]. These negative binding energies suggest a favorable interaction between donepezil and AChE, indicating a strong binding affinity.

Nevertheless, the NEP-induced inhibitory effect on the enzyme confirms the reports of Pompermaier and colleagues [35], who showed that DβH increases AChE activity in zebrafish larvae. In addition, Lisman’s 2017 world patent suggested that DβH inhibitors could be useful for some problems associated with neurodegenerative diseases, particularly memory loss [36]. In the latter case, however, attention has been focused on the role of DA, whose loss in neurodegenerative diseases leads to profound memory deficits in a process called long-term potentiation (LTP) [36]. Nevertheless, further in vivo studies should be conducted to determine NEP-induced activity and the precise mechanism of action in both the early and late stages of Alzheimer’s disease.

### 2.4. NEP Affects MRF-Induced Analgesia and Tolerance

DβH inhibition resulted in an increase in dopamine (DA) levels, while this monoamine’s involvement in pain control has been well documented over the years [37]. In this context, some studies reported that DA, in particular mesolimbic dopamine activity, is impaired in chronic pain [38,39], which in turn negatively affects motivated behavior [40]. Moreover, Taylor et al. found that microinjection of the selective D2-like dopamine receptor agonist quinpirole inhibits formalin-induced persistent nociception in the rat hind paw [41]. This was also confirmed by others [42,43]. However, some studies indicated opposite data. A good example is the study of Wood et al. [44], who showed that striatal dopamine release positively correlated with the magnitude of perceived pain. Taken together, it was demonstrated that painful stimulation could regulate dopamine signaling differently, and that dopamine could exert different effects on pain regulation depending on the pain model and the type of dopamine receptor binding [45].

Hence, our purpose was to determine whether or not NEP can affect the pain process through its involvement in increasing DA levels by preventing its conversion to NA.

**Figure 3:**
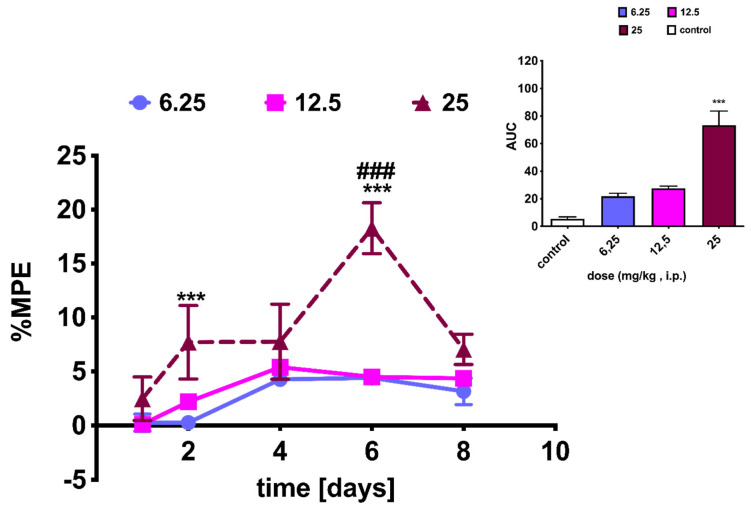
Time- and dose-dependent analgesic effect of NEP administered intraperitoneally to rats at three different doses of 6.25, 12.5, and 25 mg/kg, as measured in the tail-flick test. The dose-response data derived from the AUC calculated after NEP treatment are shown as insets in the right panel. Results were analyzed using either the two-way ANOVA followed by a Bonferroni post-test (dose-response curves) or the one-way ANOVA followed by a Dunnett post-hoc test (AUC calculation). Significant differences were found between animals receiving NEP (25 mg/kg i.p.) vs. NEP (6.25 mg/kg) on day 2 of treatment (*** *p* < 0.001), NEP (25 mg/kg i.p.) vs. NEP (6.25 mg/kg) on day 6 of treatment (*** *p* < 0.001), and NEP (25 mg/kg i.p.) vs. NEP (12.5 mg/kg) on day 6 of treatment (^###^ *p* < 0.001), respectively; *n* = 6 rats/group. In the case of AUC data, significant differences were as follows: *** *p* < 0.001, as compared to those of the control (methylcellulose-treated animals).

In this study, male rats were administered three doses of NEP (6.25, 12.5, and 25 mg/kg i.p.). However, we did not determine the effect induced by simultaneous administration of NEP (25 mg/kg i.p.) and MRF (10 mg/kg i.p.), as significant side effects occurred with chronic administration of the highest dose of NEP (visible abnormalities of motor behavior such as crawling and immobility). In addition, at the 25 mg/kg NEP dose, the period of administration of the drug was reduced to 8 days in order to not prolong the potential suffering of the animals (Figure 3). However, as can be seen in Figure 3, a time-dependent [*F*_4,60_ = 11.54; *p* < 0.0001] effect of treatment [*F*_2,15_ = 15.11; *p* = 0.0003] between the different NEP doses was revealed by two-way ANOVA. Although 25 mg/kg NEP proved to be the most effective in relieving pain, with a maximum analgesic effect at day 6, amounting to %MPE = 18.267 ± 2.367, the effect already showed a tendency to decrease after this time, reaching an insignificant effect compared to that produced by the lower doses of NEP (i.e., %MPE = 7.034 ± 1.404 for NEP 25 mg/kg vs. %MPE = 3.157 ± 1.215 and 4.354 ± 0.230 for NEP 6.25 mg/kg and NEP 12.5 mg/kg, respectively; Figure 3).

**Figure 4:**
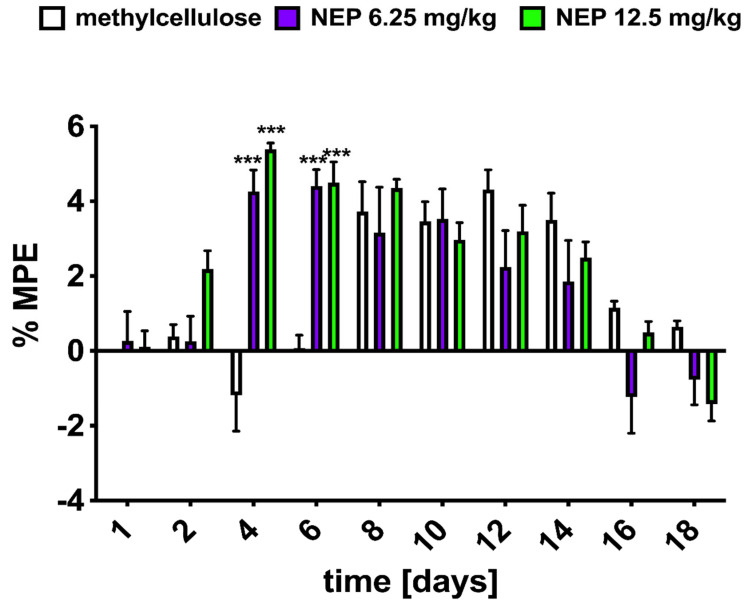
Dose- and time-response curves reflecting analgesic properties of NEP following intraperitoneal (i.p.) administration in response to noxious thermal stimuli, as measured by the tail-flick test in comparison to those of the control. NEP was administered at doses of 6.25 and 12.5 mg/kg for 17 consecutive days, and the effect was measured immediately after administration. Results from every second day, starting from day 2, were omitted. Results are presented as a percentage of maximum possible effect (%MPE) ± S.E.M. The two-way ANOVA followed by Bonferroni’s post-test showed significant differences at days 4 and 6 of treatment between NEP (6.25 mg/kg) vs. control and NEP (12.5 mg/kg) vs. control (*** *p* < 0.001); *n* = 6 per group.

In the radiant heat tail-flick test, NEP at doses of 6.25 and 12.5 mg/kg i.p. showed a time-dependent analgesic effect [*F*_9,135_ = 23.72; *p* < 0.001, *n* = 6], which reached a maximum on the sixth (%MPE = 4.404 ± 0.437 for the 6.25 mg/kg dose) and fourth day of compound application (%MPE = 5.386 ± 0.168 for the 12.5 mg/kg dose). However, there was no significant effect of treatment [*F*_2,15_ = 1.59; *p* > 0.05, *n* = 6], as both doses used behaved similarly. Furthermore, there was no difference between the test group and the methylcellulose-treated animals, suggesting that NEP was a compound with a low analgesic potential (Figure 4).

Since NEP prevents the conversion of DA to NA, its use could serve as an effective means of restoring dopamine signaling and thus influencing pain. Unfortunately, as can be seen in Figure 3 and Figure 4, NEP has no significant analgesic effect in animals exposed to thermal stimuli. These results are compatible with data from other studies [46], which reported that neither the increase in synaptic dopamine levels nor the depletion of DA amino acid precursors such as phenylalanine (Phe) and tyrosine (Tyr) had any effect on the perception of thermally induced pain.

In the case of repeated drug administration, it is well known that the midbrain dopamine system plays a key role in reward-related behaviors. Indeed, for example, chronic exposure to opioids increases dopamine release in the striatum [47]. It is also known that D2DR protein expression is increased in a time-dependent manner following chronic morphine treatment [48]. Furthermore, antagonization of the D2DR receptor, but not the D3DR receptor, was reported to attenuate morphine tolerance [48]. On the other hand, opioids suppress the release of NA by the locus ceruleus (LC) neurons [49], leading to hypotension, slowed breathing, etc. Hence, an obvious link to DBH and thus NEP exists.

**Figure 5:**
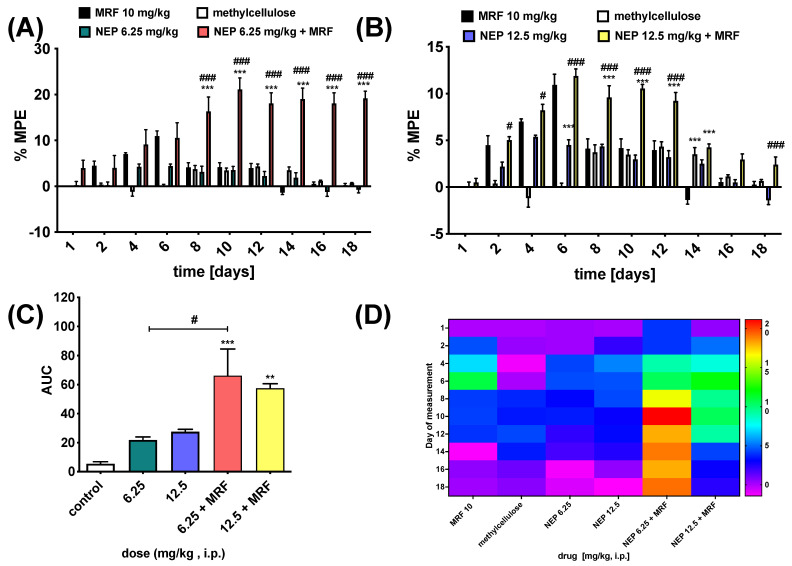
Comparison of analgesia response mediated by NEP and NEP + MRF administered intraperitoneally (i.p.) in the radiant heat tail-flick test. NEP was administered either at a dose of 6.25 mg/kg (**A**) or 12.5 mg/kg (**B**) for 17 consecutive days, either alone or with a constant dose of MRF (10 mg/kg i.p.). (**C**) represents the AUC results. (**D**) presents a created heatmap. Two-way ANOVA with Bonferroni post-hoc test was used to determine differences between treatment groups at each time point (days), as demonstrated in (**A**,**B**). The one-way ANOVA followed by a Dunnett post-hoc test was used in the AUC calculation. ^#^ *p* < 0.05 and ^###^ *p* < 0.001 indicate a significant difference between NEP and NEP + MRF (**A**,**B**), while *** *p* < 0.001 for NEP + MRF to MRF or NEP to MRF (**A**,**B**). With AUC data (**C**), the significant differences were as follows: ** *p* < 0.01 and *** *p* < 0.001, as compared to that of the control, and ^#^ *p* < 0.05 for NEP (6.25) vs. NEP (6.25) + MRF; *n* = 6 animals/group.

In this context, repeated simultaneous i.p. administration of NEP and MRF for 17 consecutive days showed that NEP significantly enhanced and prolonged morphine-induced analgesia, with a concomitant positive effect on MRF tolerance (Figure 5). However, no significant differences were found between the individual doses of NEP and MRF administered alone. Nevertheless, as compared to MRF, the addition of either the 6.25 mg/kg or 12.5 mg/kg dose of the compound resulted in a significant time- [*F*_9,135_ = 23.92; *p* < 0.0001, *n* = 6—NEP at a dose of 6.25 mg/kg, and *F*_9,135_ = 67.41; *p* < 0.0001, *n* = 6—NEP at a dose of 12.5 mg/kg] and treatment-dependent effect [*F*_2,15_ = 22.33; *p* < 0.0001, *n* = 6—NEP at a dose of 6.25 mg/kg, and *F*_2,15_ = 73.53; *p* < 0.0001, *n* = 6—NEP at a dose of 12.5 mg/kg].

Intriguingly, as shown in Figure 4, the simultaneous administration of MRF and NEP at a dose of 6.25 mg/kg resulted in a stronger antinociceptive effect (Figure 5A) when compared to the higher dose of the drug, as the %MPE value calculated for the above dose was reached on day 10 and was %MPE = 21.138 ± 2.480, while the %MPE for the dose of 12.5 mg/kg NEP was 11.888% ± 0.739% (on day 6 of drug administration; Figure 5B). In addition, the lower dose of NEP co-administered with MRF appeared to produce a similar degree of analgesia by the end of the study (MPE ≈ 19%). Whilst a bell-shaped curve appeared for the mixture of 12.5 mg/kg NEP + MRF (Figure 5B), with the maximum peak reached on day 6 post-administration. The observed effect suggests a non-dose-dependent mechanism of action for NEP when combined with morphine (MRF). Several potential mechanisms could explain this observation, including: (i) a ceiling effect of NEP, and (ii) overactivation of the dopamine system. Regarding the latter, both NEP and MRF increase dopamine levels, and their combined effects at higher doses could lead to dopaminergic overload, triggering negative feedback mechanisms or dopamine receptor desensitization. This could explain why the combination of 6.25 mg/kg NEP with MRF produces a better effect than the 12.5 mg/kg dose. Notably, excessive dopamine activity can diminish analgesia, further supporting the idea that balance in dopamine signaling is crucial for optimal pain relief.

The results obtained are crucial as they confirm the recently published data on the involvement of DA and dopaminergic neurons, particularly in the ventral tegmental area (VTA), in tolerance to morphine analgesia [50]. Furthermore, it was also shown that morphine-tolerant rats exhibited increased DA release [51]. In our case, this DA level appears to be high, considering the absence of its conversion to NA due to the activity of NEP. Noteworthy, DA is a neurotransmitter acting at two subtypes of DA receptors, namely D1-like and D2-like ones, which behave differently when considering their influence on cAMP levels [52]. This, in turn, has different consequences in terms of analgesia. Interestingly, activation of D2DR receptors in the VTA and spinal cord both produces analgesic effects, while agonists at D2DR can additionally prevent morphine-induced tolerance development [53,54]. This is compatible with our results.

Overall, the results obtained clearly indicate that, in order to delay opioid-induced tolerance, in particular, NEP might serve as a useful medical tool in the treatment of patients with severe or chronic pain who are administered opioids. Importantly, as NEP, administered simultaneously with MRF, resulted in increased pain alleviation, this compound should be considered a potent co-analgesic in the analgesic ladder. However, studies on the adverse effects resulting from the use of such a combination, both in terms of their quality and intensity, should be continued. Additionally, the behavior of NEP regarding its potential interactions with neurotransmitters involved in opioid-induced pain modulation (such as norepinephrine, serotonin, and opioid peptides) should be evaluated using selective antagonists, followed by neurochemical measurements. This approach will enable a more direct evaluation of how these neurotransmitters contribute to the observed synergistic analgesic effects induced by the concurrent administration of NEP and MRF.

## 3. Materials and Methods

**Figure 6:**
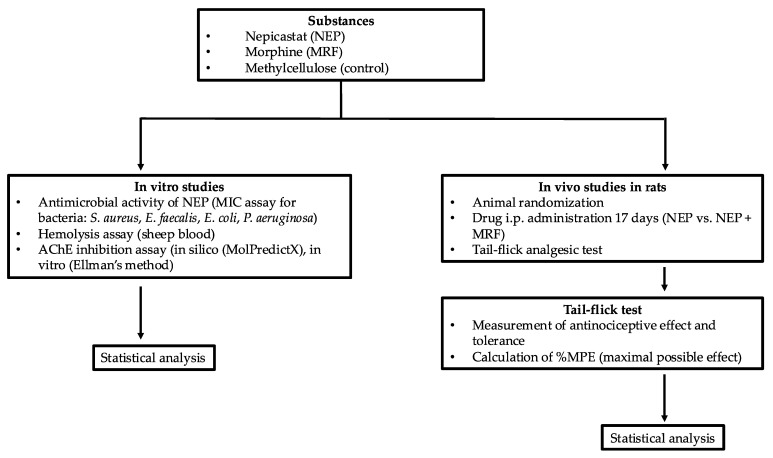
Research methodology flowchart.

As the paragraph outlines the various methods used in the study, a brief flowchart (Figure 6) has been provided to visually summarize the steps involved, aiding in the understanding of the techniques employed.

### 3.1. Chemicals, Microorganisms, and Cell Cultures

Nepicastat (NEP) was supplied by one of the co-authors (Prof. Wojciech Kamysz), morphine (MRF) was purchased from Polfa Warszawa (Polfa Warszawa, Warsaw, Poland), while methylcellulose used in behavioral in vivo studies was purchased from Sigma Aldrich (Sigma Aldrich, St. Louis, MO, USA).

In the NEP-induced antimicrobial activity test, the test microorganisms included two Gram-negative strains: *Escherichia coli* ATCC 25922 and *Pseudomonas aeruginosa* ATCC 15442, and two Gram-positive strains: *Staphylococcus aureus* ATCC 6538 and *Enterococcus faecalis* ATCC 29212. Bacterial strains were obtained from the American Type Culture Collection (ATCC). The strains were subcultured on fresh, appropriate Brain Heart Infusion Broth (Biomaxima, Lublin, Poland) agar plates 24 h prior to the antimicrobial test.

For in vivo studies, all drugs were freshly prepared on the day of the experiment and administered intraperitoneally (i.p.). MRF was dissolved in 0.9% NaCl, whereas NEP was suspended in a 0.1% solution of methylcellulose. In all experiments, the control group received 0.1% methylcellulose.

### 3.2. Determination of Minimum Inhibitory Concentrations (MICs)

The minimum inhibitory concentration (MIC) was evaluated by the two-fold microdilution method according to the CLSI reference protocol. The bacteria were maintained on a brain heart infusion (BHI) agar at 37 °C for 24 h. Bacterial inoculum was prepared in a sterile saline solution and diluted in Mueller-Hinton II broth to a final concentration of 10^6^ colony-forming units per mL (CFU/mL). Concentrations of the tested compound varied from 0.05 to 512 μg/mL. Ciprofloxacin was used as a reference over a concentration range of 0.004–4 μg/mL. Plates were incubated for 18 h at 35 °C. The lowest concentration of compounds that inhibited the visible growth of bacteria was considered the MIC. All tests were performed in triplicate.

### 3.3. Hemolysis Assay

The hemolysis assay was performed according to a protocol published elsewhere [55]. Briefly, defibrinated sheep blood was purchased from Graso (Graso Biotech, Jabłowo, Poland). Blood samples were centrifuged at 2500 rpm for 10 min (RT). The supernatant was carefully removed without disturbing the red blood cell (RBC) pellets. Samples of NEP were resuspended in 5 mL of PBS (pH 7.4, RT) and centrifuged at 2500 rpm for 10 min. The washing step was repeated three times until the supernatant was clear. After a final wash, the remaining RBC pellet was diluted 1:50 in PBS (pH 7.4, RT) to obtain a 2% RBC suspension. The positive control (100% hemolysis) was prepared with distilled water, while the negative control (0% hemolysis) was prepared with PBS (pH 7.4). Next, a 2% RBC suspension was incubated with serial dilutions of the test compounds (0.5–1 mg/mL) in a 1:1 ratio for 1 h at 37 °C. Samples were centrifuged at 4000 rpm for 5 min (RT), and 100 µL of supernatant from each sample was transferred to a 96-well plate. The supernatant was collected for detection. Optical density (OD) was measured at 540 nm in a microplate reader (BioTek Synergy HTX, Warsaw, Poland). The value of compound-induced hemolysis was calculated according to the following equation:

Hemolysis [%] = (A − Ab)/(A100% − A0%) × 100%,

where: A—absorbance of the sample incubated with compound, Ab—absorbance of blank sample, A100%—absorbance of reference (positive control), A0%—absorbance of 1% hematocrit incubated with PBS (negative control).

### 3.4. Activity Against AChE Enzyme

#### 3.4.1. Computational-Aided Prediction of NEP Biological Activity

In order to preliminarily determine AChE as another molecular target for NEP, we used a freely available online tool, MolPredictX (https://www.molpredictX.ufpb.br/; accessed on 13 March 2025) [56]. For prediction, the structure of NEP was converted to SMILE code.

#### 3.4.2. In Vitro AChE Inhibition Assay

A popular method for determining acetylcholinesterase activity is the spectrophotometric approach of Ellman et al. [57], which uses thiocholine esters as substrates and 5,5′-dithiobis-(2-nitrobenzoic acid) (DTNB) as the thiol reagent. It is based on the interaction of thiocholine, which is produced by the enzymatic hydrolysis of a synthetic substrate, acetylthiocholine (ATCh), with DTNB or Ellman’s reagent. The yellow product of this reaction, 5-thio-2-nitrobenzoic acid (TNB), is produced by measuring absorbance at 412 nm. The activity of acetylcholinesterase was measured using Ellman’s method [57], which was slightly modified and previously documented in an earlier study [58]. In short, 1.6 mL of 100 mM of phosphate buffer pH (7.5), 0.1 mL of 4 mM of 5,5′-dithio-bis-2-nitrobenzoic acid (DTNB), and 0.1 mL of NEP at concentrations of 20 mg/mL, 10 mg/mL, 2 mg/mL, and 0.2 mg/mL were all immediately dissolved in 2 mL of the reaction mixture to create the reaction mixture (final volume of 2 mL). By measuring absorbance at 412 nm after two minutes of incubation at 25 °C, the method was based on the formation of a yellow anion, 4,4′-dithio-bis-acid nitrobenzoic acid. Two minutes were spent during the pre-incubation of the enzyme. Acetylthiocholine iodide (0.1 mL, 200 mM) was added to initiate the reaction.

### 3.5. Behavioral Studies

#### 3.5.1. Animals

A total of 42 male Wistar rats (Bialystok, Poland), weighing 200–250 g, were used for the pain-relieving effect. The number of animals required for the behavioral experiments was calculated as 6–8 rats per group. Ultimately, animal groups consisted of 6 per group. Animals were maintained in a temperature-controlled room (22 ± 2 °C) and humidity (55% ± 5%), under a standard 12 h light-dark cycle, with free access to water and food.

Animals were habituated to the method and experimental apparatus before the day of measurements. Each animal was used only once and was sacrificed immediately after the experiment.

All experimental procedures using animals complied with the policies on the care and use of laboratory animals published in the European directive 2010/63/EU and were approved by the 1st and 2nd Local Commissions for the Care and Use of Laboratory Animals for Experimental Procedures (permission no. 12/2015, 512/2018). The entire experimental design was based on the rule of the replacement, refinement, and reduction (the 3Rs) to reduce the suffering of the animals and use the minimum number of animals.

#### 3.5.2. Analgesia and Tolerance Induced by Morphine in Rats and NEP Influence

In order to evaluate the pain-relieving effect exerted by intraperitoneal (i.p.) NEP, the drug was administered subacutely (within 17 days) to rats at three different doses (6.5, 12, 25 mg/kg). Additionally, a co-injection of NEP with a constant dose of MRF (10 mg/kg i.p.) was performed over 17 days, so NEP’s impact on morphine analgesia and tolerance was determined. Antinociceptive responses to either NEP alone or NEP + MRF were determined by the tail-flick test as described below. The results were compared to those obtained for morphine (as a positive control; 10 mg/kg i.p.) and methylcellulose (0.1%), respectively.

##### Tail-Flick Test

Antinociceptive responses to drugs were determined by the tail-flick test as described elsewhere [20]. Briefly, rats were placed in a radiant heat automatic tail-flick analgesymeter (Ugo Basile, Gemonio, Italy), and the reaction latencies (tail-flick latency; TFL) were measured. The reaction time of animals to radiant heat was recorded by exposing the tail (2–3 cm from the tip) to a light beam. The tail withdrawal from the radiant warmth was taken as an endpoint. The cut-off time of 15 s was used to avoid the animal’s tail injury.

On the first day of the study (day 0), the basal analgesic (pre-drug) latency was determined for all animals (baseline value). Next, TFLs were recorded every day prior to drug administration. TFL was elicited thrice every day in each laboratory animal at 5 s intervals. Day 18 was the only time when measurements were made and no drug treatment was performed. All measurements of analgesia were carried out at the same time each day.

Nociceptive data (TFL) were expressed as the percent maximum possible effect (%MPE) using the formula: %MPE = [(test latency − baseline)/(cut-off − baseline)] × 100.

### 3.6. Statistical Analysis

In vitro experiments: the results were presented as the mean ± SEM. One-way ANOVA followed by Tukey or Bonferroni post-tests was used in order to determine any statistical differences. *p* < 0.05 was considered statistically significant.

In vivo experiments: animal groups consisted of 6 per group. In addition, block randomization was used to allocate animals into their respective treatment groups. The same investigator evaluated the behavioral studies in all groups and was unaware of the treatment given. In the behavioral studies, the Bonferroni post-hoc test was used following a two-way repeated-measures ANOVA to analyze the differences between the experimental groups at individual time points (days). The area under the curve (AUC) was analyzed by using a one-way ANOVA followed by Dunnett’s post-hoc test. All data in the study are expressed as the mean ± standard error (SEM), and the significance level was set at *p* < 0.05.

All statistical analyses and figure preparations were performed using the GraphPad Prism software package (version 9.00 for Mac, GraphPad Software, San Diego, CA, USA, www.Graphpad.com).

## 4. Conclusions

Nepicastat (NEP) was presented as a multifactorial agent with a broad spectrum of activities. These create new possibilities for using NEP, as well as its potential analogues, as useful targets for medication treatment. Nevertheless, considering the importance of NA being the product of DβH activity, the NEP compound can also have adverse effects. Therefore, further studies should be performed, which will be focused mainly on its safety profile, with particular emphasis on research conducted into the characterization of the drug as a provocative agent of other diseases.

## Figures and Tables

**Table 1:** The minimal inhibitory concentration (MIC) [%] of NEP [µg/mL].

Tested Bacteria				
	*Staphylococcus aureus* ATCC 6538	*Enterococcus faecalis* ATCC 29212	*Escherichia coli* ATCC 25922	*Pseudomonas aeruginosa* ATCC 15442
Nepicastat (NEP; µg/mL)	64	128	128	>521
Ciprofloxacin (ctrl; µg/mL)	0.125	1.0	0.008	0.06

**Table 2:** The predicted activity of NEP towards AChE enzyme by an online tool MolPredictX.

Acetylcholinesterase (AChE)				
Predicted outcome	Probability	Probability active	Probability inactive	Predicted reliability
Active	0.6	0.6	0.4	reliable

## Data Availability

The data presented in the study are available upon request from the corresponding author.
